# Face Privacy Protection Method for Autonomous Sensors Based on Hierarchical Format-Preserving Encryption

**DOI:** 10.3390/s25237369

**Published:** 2025-12-03

**Authors:** Haojie Ji, Long Jin, Junjie Zhang, Te Hu, Chongshi Xin, Yuchi Yao

**Affiliations:** 1 Key Laboratory of Modern Measurement & Control Technology, Ministry of Education, Beijing Information Science & Technology University, Beijing 100192, China; jihaojie@bistu.edu.cn (H.J.); 2023020004@bistu.edu.cn (T.H.); 2023020128@bistu.edu.cn (C.X.); 2024020009@bistu.edu.cn (Y.Y.); 2 College of Mechanical and Electrical Engineering, Beijing Information Science & Technology University, Beijing 100192, China; 3 Hefei Innovation Research Institute of Beihang University, Hefei 230012, China; zhangjunjie55@163.com

**Keywords:** face privacy, data security, format-preserving encryption, functional usability, pedestrian detection

## Abstract

Advanced sensors in connected automated vehicles (CAVs) increasingly collect facial biometric information for environmental perception, posing serious privacy leakage risks. However, existing privacy protection methods for automotive data primarily focus on strict security mechanisms and fail to fully balance data usability. This paper presents a hierarchical format-preserving encryption (H-FPE) method for face privacy protection in autonomous sensors. The proposed method constructs a privacy-preserving framework for face detection based on YOLOv11 by employing a region-specific encryption strategy where the encryption strength is tailored to the importance of different facial regions. The encryption algorithm employs SM4-based Feistel structures with pseudo-random functions to ensure RGB value constraints while maintaining image format integrity. Experimental evaluation results in diverse scenarios demonstrate that the proposed privacy encryption method achieves superior privacy protection performance. In terms of encryption strength, the method achieves entropy efficiency exceeding 98%, with an average entropy increase of 0.77 bits, representing an improvement of approximately 9.4% over the traditional thumbnail-preserving encryption (TPE) method. Considering the usability of downstream tasks, the proposed method preserves pedestrian detection performance, with F1-scores exceeding 97% in selected scenarios, demonstrating a 0.5% difference compared to TPE while providing substantially stronger privacy protection. The H-FPE method effectively balances privacy protection and functional usability, offering a robust solution for facial data protection in autonomous sensor applications while preserving essential detection capabilities.

## 1. Introduction

CAV technology plays an increasingly critical role by using autonomous sensors for environmental perception, pedestrian detection, and traffic safety monitoring [1,2,3]. CAVs are equipped with numerous visual sensors capable of real-time acquisition and processing of image data containing facial information from road environments, providing essential data support for autonomous driving, pedestrian recognition, and safety warning systems. However, facial data continuously collected and transmitted by sensor networks contains rich biometric information that is inherently privacy-sensitive. Unauthorized disclosure or malicious exploitation of such data may lead to severe privacy violations, including identity theft, location tracking, and behavioral profiling [4,5]. In the domain of intelligent transportation system (ITS), facial data should support downstream computer vision tasks such as pedestrian detection and object recognition for critical applications, even after privacy-preserving processing. The existing data encryption methods typically employ complete encryption strategies that effectively protect data privacy while compromising the visual features and structural information of images. This fundamental tension between privacy protection and downstream tasks’ utility constitutes a core technical challenge in the field of CAVs [6].

The facial privacy protection techniques have typically evolved along several distinct developmental trajectories. Traditional privacy-preserving methods include data anonymization [7,8], differential privacy [9,10], and homomorphic encryption [11,12]. While demonstrating excellent privacy protection capabilities, these techniques typically eliminate the utility of image data for downstream computer vision tasks. In recent years, format-preserving encryption (FPE) has emerged as a promising privacy protection approach, with its core advantage lying in preserving the format characteristics of original data while encrypting. In the domain of image privacy protection, existing research primarily focuses on full-image encryption and local obfuscation techniques [13,14]. Despite offering strong security, full-image encryption methods completely obscure visual features and structural information, thereby preventing the execution of downstream tasks such as pedestrian detection. Comparatively, local obfuscation and pixelation methods preserve the overall image structure but exhibit insufficient security against sophisticated attacks. Although deep learning-based adversarial perturbation methods could achieve a better balance between privacy protection and data utility, the robustness and generalization capabilities remain debatable. The development of TPE technology provides a promising direction to address this challenge. However, existing TPE algorithms and ideal TPE algorithms offer specific privacy protection capabilities while maintaining data format, yet they remain limited in encryption strength and data availability [15,16]. Particularly, most of the current methods fail to concurrently meet the dual requirements of strong privacy protection and high-performance downstream computer vision tasks.

In CAV scenarios, facial data faces various privacy threats. Eavesdroppers may intercept images during transmission. Server insiders with legitimate access may attempt unauthorized identity extraction. External attackers may deploy facial recognition models for re-identification. Consistent with Kerckhoffs’s principle, it is assumed that adversaries have complete knowledge of the encryption algorithm and access to encrypted images, yet remain unaware of the secret key. Within this threat model, potential attacks include statistical analyses such as frequency and correlation analysis. To address these threats, effective privacy protection must provide strong protection in sensitive facial areas while retaining structural information in boundary areas for detection utility. Additionally, the method must meet CAV operational requirements, enabling encrypted images to support pedestrian detection training for safety-critical functions such as collision avoidance and pedestrian tracking.

In order to address the contradiction between facial data privacy protection and data availability in CAVs, this paper proposes an H-FPE method, with the core objective of maximizing the preservation of downstream tasks’ performance while providing privacy protection for facial regions. The main contributions of this work are summarized as follows.

First, a distance transform-based hierarchical encryption strategy is proposed for facial region protection. The strategy employs the Euclidean distance transform to compute the spatial distance from each pixel to the nearest facial region boundary. Based on these computed distance values, the facial region is intelligently partitioned into edge zones and core zones. Subsequently, different encryption intensities are applied to preserve facial data according to the privacy sensitivity and perceptual importance of each zone. Specifically, low-bit encryption is used in the edge zones to preserve contour features essential for object detection tasks, while full-bit encryption is used in the core zones to ensure robust protection of identity information. This hierarchical approach overcomes the limitation of conventional methods that apply uniform encryption across entire facial regions, thereby establishing a principled framework for optimizing the trade-off between privacy protection and visual task utility.

Second, a Feistel-based FPE algorithm is designed integrating SM4, a national cryptographic standard. The algorithm constructs a multi-round iterative encryption mechanism through the Feistel network architecture. Pseudo-random functions and modular arithmetic operations are applied to ensure that encrypted RGB pixel values remain within the valid range of 0 to 255. This Feistel-based FPE design maintains full compatibility with standard image processing pipelines. In addition, the Feistel structure provides mathematical guarantees for cryptographic invertibility, enabling lossless decryption under proper authorization. This work presents a position-dependent tweak mechanism that binds encryption outputs to spatial coordinates, ensuring that identical pixel values at different locations yield distinct ciphertexts and thereby significantly enhancing resistance against frequency analysis and statistical correlation attacks.

Finally, a dual-metric evaluation framework is presented, quantifying both privacy protection strength and functional utility. Privacy strength is assessed through information-theoretic metrics, including Shannon entropy and its derivatives, while functional utility is measured through performance metrics on downstream computer vision tasks, specifically pedestrian detection accuracy and recall rates. This framework provides rigorous quantitative benchmarks for evaluating privacy-preserving methods and facilitates systematic comparison across different algorithms. Experimental validation demonstrates that the proposed method achieves a superior balance between privacy protection and task utility compared to existing approaches.

## 2. Literature Review

Facial privacy protection has emerged as a critical frontier in computer vision and information security. Balancing effective privacy protection with system functionality has become an urgent challenge. In response, a variety of technical solutions have been proposed, which can be broadly categorized into four main strands, i.e., anonymization-based methods, adversarial perturbation-based methods, encryption-based methods, and generative model-based methods. This section reviews their core principles, characteristics, and limitations.

### 2.1. Anonymization-Based Methods

As the earliest and most intuitive technical approaches, the anonymization-based privacy protection methods prevent identity recognition by reducing image quality or occluding key regions, with the primary techniques including pixelation, Gaussian blurring, and facial feature masking.

Currently, the most commonly used anonymization methods are k-same series algorithms. Newton et al. pioneered the application of k-same methods to address facial privacy protection in images by introducing the concept of k-anonymity to achieve privacy protection [17,18]. The approach first clustered images into at least k images in each cluster, computed the average face image for each cluster, and then replaced original images with average face images to complete anonymization. This method provided privacy protection with a de-identification rate of at most 1/k, while the visual quality of the resulting average faces needed to be improved due to pixel-level superposition of k images. Subsequently, numerous improvements to the k-same method have been proposed, e.g., K-Same-Eigen [19], K-Same-M [20], and K-Same-Select [21] methods. Gross et al. proposed the K-Same-Select method, which introduced additional control information during the anonymization phase, such as non-identity attributes like skin tone and makeup. This method not only achieved privacy protection but also improved the utility of anonymized data. Gross et al. further proposed the K-Same-M method by introducing the active appearance model (AAM) and applied k-anonymity to complete facial image anonymization. Since this method is operated at the model parameter level rather than at the pixel level, the image quality of the anonymized results is significantly improved. Although k-same series algorithms consider both privacy protection and data utility, they still present notable limitations. For instance, the generated anonymized images lack natural visual appearance, and the degree of data utility remains insufficient.

Overall, anonymization-based privacy protection methods suffer from fundamental limitations. First, these methods achieve privacy protection through information loss or blurring, resulting in irreversible processing, making them unsuitable for authorized requirements of original data. Second, k-same series algorithms require numerous similar samples for clustering and averaging, making it difficult to meet response time requirements in real-time processing scenarios for CAVs. Third, blurring and pixelation severely destroy image detail features. While preventing identity recognition, they also lead to significant performance degradation in computer vision tasks such as pedestrian detection and object tracking, thereby failing to meet the functional requirements of onboard safety systems for images. Fourth, these methods lack precise control over the degree of privacy protection, often either providing insufficient protection leading to identity leakage or causing a complete loss of functionality through excessive processing. Therefore, anonymization methods show clear inadequacies in autonomous driving that require simultaneous guarantees of privacy protection and system functionality.

### 2.2. Adversarial Perturbation-Based Methods

Adversarial sample techniques exploit the vulnerability of deep neural networks by adding carefully designed small perturbations to images, thereby deceiving facial recognition models. This method has emerged as a promising privacy protection approach in recent years. The core idea of this method is to construct adversarial samples that exploit the fragility of deep learning models while maintaining image visual quality, causing recognition models to make incorrect judgments.

Sharif et al. proposed a physical-world privacy protection scheme based on adversarial eyeglasses [22]. The method optimized patterns on eyeglass frames to cause misidentification by facial recognition systems, and demonstrated that adversarial samples could transfer from the digital domain to the physical world. This work provided a theoretical foundation for wearable privacy protection devices. Komkov and Petiushko further investigated adversarial hat and adversarial sticker techniques. These methods interfere with the recognition process by attaching or wearing special patterns at specific facial locations [23]. The advantage of such physical adversarial methods is real-time privacy protection for users without requiring image post-processing.

In order to improve the robustness and transferability of adversarial perturbations, researchers have proposed improved algorithms. Dong et al. proposed the momentum iterative fast gradient sign method (MI-FGSM), which introduced momentum terms in the gradient update process to enhance the transferability of the generated adversarial samples across different recognition models [24]. To address privacy protection in black-box scenarios, researchers have explored generation methods for universal adversarial perturbations that aim to construct perturbation patterns effective against multiple recognition systems.

However, adversarial perturbation-based privacy protection methods have inherent limitations. First, adversarial perturbations heavily depend on the structure and parameters of target recognition models. When recognition systems are updated or employ defense mechanisms, existing perturbations may become ineffective, leading to privacy protection failure. Second, adversarial perturbations essentially exploit model vulnerabilities rather than providing data-level protection. They may be eliminated by adversarial sample detection techniques or image preprocessing methods such as JPEG compression and Gaussian filtering. Third, these methods lack quantifiable privacy protection guarantees, with no assurance of effectiveness across all unknown recognition systems. Finally, physical adversarial methods require users to actively wear specific devices, which may be inapplicable or inconvenient in certain scenarios. For the scenarios with long-term stable operation and facing diverse recognition threats, privacy protection schemes relying on adversarial perturbations struggle to meet requirements for sustained effectiveness and universality.

### 2.3. Encryption-Based Methods

Encryption technology provides the strongest security guarantee for facial privacy protection from a cryptographic perspective. These methods can be subdivided into traditional image encryption and FPE branches.

Li et al. designed a facial recognition privacy protection method based on homomorphic encryption [25]. This method first used the popular authentication model FaceNet to extract user biometric information, and then employed ring learning with errors (RLWE)-based homomorphic encryption to encrypt the extracted feature information. This work ensured that biometric information remained privacy data when outsourced to servers for distance computation, preventing servers from snooping on user behavior. In addition, this method presented the concept of random numbers in the authentication process to prevent replay attacks by unauthorized users against servers.

Alsafyani et al. proposed a revolutionary facial feature encryption technique that combined image optimization with cryptography and deep learning architectures [26]. To strengthen key security, optical chaos mapping was employed to manage the initial standards of a five-dimensional conservative chaos method. In addition, secure encrypted generative adversarial networks and chaotic optical maps were provided to complete the encryption and decryption process of facial images. The target field served as a hidden factor in the machine learning method within the encryption approach. Modern networks are used to restore encrypted images to unique images for image decryption. A region of interest (ROI) network is provided to extract relevant items from encrypted images for easier data mining in privacy-preserving settings.

Zhang et al. proposed a chaotic color multi-image compression encryption and least significant bit (LSB) data-type steganography scheme [27]. In their approach, they first generated a series of chaotic sequences through iterative chaotic maps. They then employed compressed sensing to compress multiple secret images, which were fused into one large composite image. This composite image was subsequently encrypted, and the encrypted result was embedded into multiple cover images through LSB steganography. The encryption process helped mask the statistical property changes caused by the steganography operation. Finally, the stego-images were applied as textures to three-dimensional (3D) object models to achieve data-type steganography for image data.

Ciftci et al. proposed two fully reversible privacy protection schemes implemented within the JPEG architecture [28]. In both schemes, privacy protection was achieved using false color. The first scheme was applicable to other privacy protection filters, while the second scheme was specific to false color, and both schemes supported lossless or lossy modes. This method was not based on ROI and could be applied to entire frames without affecting clarity. At a result, this method relieved users from defining ROIs and improved security, as ROI tracking may fail under dynamic content, thereby exposing sensitive information.

Although encryption technology provides strong security guarantees in theory, existing methods still face key challenges in CAVs. Traditional image encryption methods such as advanced encryption standard (AES) and chaotic encryption offer high security. However, encrypted data completely loses original image structure and format characteristics. It cannot be recognized by standard image processing tools or support computer vision tasks such as object detection and pedestrian recognition. This leads to a complete loss of system functionality. Homomorphic encryption supports ciphertext-domain computation but has extremely high computational complexity, making it difficult to meet the real-time requirements of onboard systems. FPE, as standardized in NIST SP 800-38G, including modes FF1 and FF3, enables encryption while maintaining the original data format. However, these standards are primarily designed for structured data and fail to account for the spatial correlations or preserve the task utility required in visual information. Traditional TPE suffers from irreversibility due to quantization errors in frequency-domain transformation, while ideal TPE, based on order-preserving encryption, achieves reversibility but has limited randomization due to order-preserving properties. Therefore, achieving an effective balance between strong privacy protection and high utility while ensuring format compatibility and full reversibility remains an urgent problem to be solved.

### 2.4. Generative Model-Based Methods

Generative model-based privacy protection methods learn latent feature representations of faces and generate new facial images that are similar to original faces in certain attributes while altering identity information, thereby achieving de-identification [29]. These methods leverage the powerful representation capabilities of deep generative models such as generative adversarial networks (GANs) and variational autoencoders (VAEs) to decouple and reconstruct identity information in the feature space.

Sun et al. proposed a GAN-based facial inpainting method that reconstructed facial regions through generative models by generating new facial features to replace original identity information [30]. This method maintained image naturalness with generated faces appearing visually realistic and credible. Li et al. investigated attribute-preserving facial de-identification techniques based on conditional generative models, which preserved non-sensitive attributes such as age, gender, and expression while changing identity [31]. This work enabled generated facial images to functionally meet certain application requirements. Maximov et al. proposed conditional identity anonymization GAN (CIAGAN), a framework that achieved controllable de-identification processing by introducing identity decoupling mechanisms [32].

The development of diffusion models has advanced their application in facial privacy protection. By generating high-quality images through progressive denoising, diffusion models provide notable advantages over traditional GANs, such as training stability and generation quality. This capability has been harnessed in several studies that employ pre-trained models like stable diffusion for identity transformation, thereby achieving facial de-identification through text guidance or latent space manipulation.

Despite excellent performance in visual effects, generative model methods still face critical challenges in privacy protection techniques. First, generated facial images are synthetically created and fundamentally differ from the original images at the pixel level, making truly reversible recovery impossible. As a result, the original facial information cannot be restored through decryption operations, thereby limiting the application scope of the method in authorized scenarios. Second, training generative models requires large-scale datasets and substantial computational resources, while their output quality is highly dependent on the distribution and diversity of training data. This dependency often leads to artifacts or distortions when processing out-of-distribution samples. Third, these methods lack precise control over the degree of privacy protection, potentially leading to excessive modification that degrades functional utility or insufficient modification that leaves residual identity information. Fourth, the generation process typically requires long inference times, making it difficult to meet real-time requirements. Finally, generative models may introduce new privacy risks, such as privacy leakage caused by model memorization of training data. These limitations make generative model methods difficult to apply directly in the driving environments where strict requirements for reversibility, real-time performance, and robustness must be met.

Table 1 compares the proposed H-FPE with existing facial privacy protection approaches. Unlike methods that compromise either format integrity, reversibility, or task utility, H-FPE achieves desirable properties through hierarchical encryption, making it particularly suitable for privacy-preserving CAV applications.

## 3. Encryption Method

This section presents the design and implementation of the hierarchical strategy-based facial privacy protection method. The method achieves intelligent hierarchical partitioning of facial regions through image feature distance transformation, and designs differentiated encryption strategies according to the privacy importance of different regions. This approach ensures stringent privacy protection for core facial features while maintaining high image structural integrity and downstream tasks’ utility.

### 3.1. Hierarchical Encryption Strategy Design

#### 3.1.1. Facial Region Hierarchical Method Based on Distance Transform

Different facial regions hold varying degrees of importance for personal identity recognition. Core feature regions, such as the eyes, nose, and mouth, contain critical biometric information and form the primary basis for facial recognition algorithms. In contrast, peripheral areas such as facial contours and transition zones, while still informative, contribute less critically. Therefore, this study proposes a region hierarchical method based on distance transformation. By quantifying the geometric relationship between pixel positions and facial boundaries, this method provides a theoretical foundation for differentiated encryption strategies in facial regions. As an important method for analyzing shape features in digital image processing, distance transformation could effectively characterize the spatial importance distribution of pixels within facial regions. Specifically, distance transformation establishes a mapping relationship between pixel positions and privacy sensitivity by computing the shortest distance from each pixel to the boundary within the facial region.

For facial mask images M(x,y), the Euclidean distance transform is defined as:

(1) $$d(x,y)=\underset{(\mathrm{x’},\mathrm{y’})\in 𝜕M}{\min}\sqrt{(x-x’{)}^{2}+(y-y’{)}^{2}},$$

where 𝜕M represents the set of boundary pixels of the facial mask. Pixels with higher distance values are typically located near the geometric center of the facial region, thereby identifying important facial features. Whereas pixels with smaller distance values are located in edge regions, primarily containing facial contour and facial feature transition information.

Considering the impact of different face sizes on hierarchical results, this study performs normalization processing on facial feature distance values:

(2) $$d_{norm}(x,y)=\frac{d(x,y)}{d_{max}},$$

where $d_{max}$ is the maximum distance value within that facial region. The normalization operation maps distance values to the [0, 1] interval, providing a benchmark for unified threshold segmentation. This processing approach ensures consistency and robustness of the proposed facial region hierarchical algorithm across different face sizes.

#### 3.1.2. Hierarchical Threshold Setting and Adjustment Mechanism

The selection of threshold parameters is the core of hierarchical strategy design, as it directly determines the division boundary between core regions and edge regions. Therefore, reasonable threshold setting needs to comprehensively consider privacy protection strength and image utility. This study establishes a dynamic threshold setting mechanism through theoretical analysis and experimental validation.

According to the image normalized distance transformation results, the facial region hierarchical rule is defined as:

(3) $$Layer(x,y)=\left\{\begin{array}{c} 1,\;if\;d_{norm}(x,y)\leq T\\ 2,\;if\;d_{norm}(x,y)>T \end{array}\right.,$$

where T is an adjustable threshold parameter. Smaller thresholds produce larger core regions and provide stronger privacy protection but may affect image structural integrity. Conversely, larger thresholds produce smaller core regions, which better preserves image features but may compromise privacy.

Through statistical analysis of numerous facial samples, this study determines the threshold value range as [0.2, 0.5]. Within this range, the core region effectively encompasses the primary features for identity recognition while maintaining an appropriate balance in regional area proportions. Considering empirical values, balancing privacy protection effectiveness and image quality, the default threshold is set to 0.3. The proposed threshold adjustment mechanism supports dynamic parameter configuration, enabling users to adjust according to specific application scenario requirements. In scenarios with high privacy protection requirements, the threshold may be reduced to expand the core region. Conversely, the threshold could be appropriately raised to preserve more structural information in the scenarios with high utility requirements.

#### 3.1.3. Edge Region and Core Region Division

Based on distance transformation and threshold segmentation results, the facial region is precisely divided into two zones with different privacy protection requirements. The edge region, located in peripheral facial areas, contains facial contours, texture transitions, and low-frequency structural information. These regions have limited importance for facial recognition but are significant for maintaining visual continuity and supporting downstream computer vision tasks. Pixels in edge regions exhibit strong inter-pixel correlations with gradual texture changes, making them suitable for lightweight encryption to maintain structural integrity. In contrast, the core region, located in central facial areas, contains critical biometric features such as eyes, nose, and mouth. These features contain rich biometric information serving as the primary basis for identity confirmation. Pixels in the core region possess high recognition value and privacy sensitivity, requiring strong encryption protection to prevent identity information leakage.

The quality of hierarchical partitioning directly affects the effectiveness of subsequent encryption. To quantitatively evaluate this quality, a hierarchical statistical analysis mechanism is established to compute metrics such as pixel count, area proportion, and distance distribution characteristics for each region. Statistical results show that the core region typically occupies 35–45% of the total facial area under the default threshold of 0.3, while the edge region occupies 55–65%. This proportional relationship conforms to the general pattern of facial feature distribution.

### 3.2. Algorithm Design

#### 3.2.1. Feistel Structure Design Based on SM4

Key management serves as the fundamental guarantee for encryption algorithm security. This study develops a complete key generation and management system based on the SM4 cryptographic algorithm, providing reliable cryptographic support for hierarchical encryption. As a block cipher standard independently designed by China, the SM4 algorithm features a 128-bit key length and demonstrates excellent security performance, making it suitable for constructing high-security encryption systems.

The key management process includes two core steps: user key standardization and round key expansion. User key standardization converts input keys of any length into a 16-byte standard format to meet SM4 requirements. For keys with insufficient length, zero padding is adopted for extension. For keys exceeding the length, truncation is adopted for compression. This processing approach ensures consistency and compatibility of key formats.

Based on the standardized master key, this paper generates 32 32-bit round keys through the SM4 key expansion algorithm:

(4) $$RK_{i}=KeyExpansion\left(K_{master},i\right),\;i=0,1,\ldots ,31,$$

The key expansion process follows SM4 standard specifications by employing a combination of nonlinear transformation and linear transformation to ensure good statistical properties of the round key sequence. Each round key is generated through complex transformations, resulting in statistically independent and unpredictable from each other.

The generated round key sequence provides rich key material for subsequent multi-round encryption operations. As key security directly affects the reliability of the entire encryption system, the adoption of the SM4 key expansion algorithm ensures a high security level for key management.

#### 3.2.2. Position-Dependent Pseudo-Random Factor Computation

To enhance the security and prevent identical pixel values at different positions from producing identical encryption results, this study designs a position-dependent pseudo-random factor generation mechanism. This mechanism fuses spatial coordinate information of pixels to generate unique perturbation values for each pixel, thus significantly improving resistance against statistical analysis attacks.

The position-dependent tweak function is defined as:

(5) $$Tweak(x,y)=(x\cdot C_{1}+y\cdot C_{2})⊕C_{3},$$

where $C_{1}$ and $C_{2}$ are prime numbers selected to be greater than 2^31^, ensuring maximum periodicity of the function. The two constants are coprime, avoiding simple linear relationships. $C_{3}$ is a pre-selected constant, and its binary representation has excellent bit balance. In the computation, the arithmetic result $\left(x\;\cdot \;C_{1}\;+\;y\;\cdot \;C_{2}\right)$ is treated as its natural 32-bit binary representation for the bitwise XOR operation.

The tweak function possesses important properties of ideal pseudo-random functions. First, it demonstrates a strong avalanche effect, where minor coordinate modifications result in significant changes in output. Second, the outputs maintain distribution uniformity across all coordinates within the image range, closely approximating a uniform statistical distribution. Finally, it displays high irregularity with no obvious correlation between tweak values at adjacent positions. By incorporating position-dependent factors, the mechanism ensures that identical pixel values at different positions yield distinct encryption results, substantially increasing resistance to statistical analysis attacks. Meanwhile, the computational overhead remains minimal, significantly preserving system performance.

#### 3.2.3. RGB Value Domain Constraint Mechanism

The principle of H-FPE is to achieve effective privacy protection while ensuring that encryption results strictly remain within the original value domain range. For image data, encrypted pixel values need to remain within the reasonable range of [0, 255].

This algorithm employs bit separation and recombination operations to achieve precise value domain control. The encryption process first separates original pixel values into preserved parts and modifiable parts. Encryption transformations are then applied to the modifiable parts, while the preserved bits remain unchanged. Finally, the modified components are recombined with the preserved bits to form the encryption output. This mechanism mathematically guarantees the validity of encrypted pixel values.

The algorithm employs a modular arithmetic mechanism to implement value domain constraints:

(6) $$P_{encrypted}=P_{preserved}\vee ((P_{mode}+Random)mod(BitMask+1)),$$

where Random is a pseudo-random value generated based on tweak and round keys. The adoption of modular operations ensures that encryption results always fall within the legal value domain range while maintaining reversibility of the encryption transformation.

### 3.3. Hierarchical Encryption Processing

#### 3.3.1. Lightweight Encryption for Edge Regions

Since edge regions primarily contain structural and textural information, excessive encryption processing would compromise their integrity, thereby affecting the performance of downstream computer vision tasks. Therefore, a lightweight encryption scheme is adopted for these regions, which provides necessary privacy protection while maximizing the preservation of image utility.

The core characteristics of lightweight encryption are reflected in three aspects: limited round number control, precise bit-level constraints, and simplified transformation operations. In terms of round number control, edge regions typically employ 2 to 4 rounds of encryption processing, significantly fewer than the 8 rounds applied to core regions. The fewer rounds could reduce computational complexity while reducing the degree of perturbation to the original pixel distribution.

Edge regions employ bit mask 0x0F, modifying only the low 4 bits of pixel values while keeping the high 4 bits unchanged. Mathematically, this constraint partitions the 256 possible pixel values into 16 equivalence classes. Pixel values within each equivalence class can convert to each other after encryption but will not cross to other equivalence classes. This design preserves the main magnitude information of pixel values, thereby maintaining the overall brightness distribution of images.

Additionally, the lightweight encryption process comprises two stages, i.e., initial confusion and multi-round key mixing. In the initial confusion stage, pixel values undergo XOR operations with position-dependent Tweak factors to establish spatial dependency. Subsequently, the multi-round key mixing stage sequentially uses different SM4 round keys for XOR operations, thereby enhancing randomness. Throughout both stages, bit mask constraints are strictly enforced to ensure precise control of the modification range.

In summary, the lightweight encryption introduces moderate visual perturbations in edge regions, eliminating precise pixel-level information while maintaining overall regional features and spatial structure. This processing approach effectively balances the requirements of privacy protection and image quality.

#### 3.3.2. Full Encryption Implementation for Core Regions

Core regions contain important identity recognition information in facial images, and therefore require high-strength full encryption protection. To ensure the complete loss of visual recognizability in these regions, the encryption needs guarantee that even when subjected to advanced analytical techniques, no useful identity information can be recovered from the encrypted result.

Full encryption employs an 8-round processing structure, with each round performing complete cryptographic transformation. Compared to lightweight processing of edge regions, full encryption has more transformation rounds, more complex operation steps, and stronger diffusion effects. The increased number of rounds significantly enhances encryption security, making it significantly more difficult to recover any useful information from the encrypted images.

The encryption process contains three main stages: initial confusion, multi-round key processing, and circular shift transformation. The initial confusion stage is similar to lightweight encryption, establishing position dependency through tweak factors. While the multi-round key processing stage uses 8 different round keys for XOR operations. The use of each round key can enhance encryption complexity. Circular left shift transformation is the main operation of full encryption, and enhances diffusion effects through bit shift operations:

(7) $$P_{shifted}=((P\ll 1)\;\vee \;(P\gg 7))\wedge 0xFF,$$

where “≪” and “≫” represent circular left shift and circular right shift operations, respectively. Circular left shift ensures that each bit position can affect other bit positions. The operation could achieve excellent bit confusion effects, and is widely used in cryptography to enhance nonlinear characteristics.

Full encryption uses 0xFF bit mask by modifying all 8 bits of pixel values. This ensures no direct numerical relationship remains between encrypted pixel values and original values, causing the encryption results to present as visually random noise. Statistical analysis shows that fully encrypted core regions have pixel histograms approaching uniform distribution, thereby meeting cryptographic security requirements.

#### 3.3.3. Differentiated Encryption Strength Control

The differentiated strength control mechanism serves as the foundation for achieving fine-grained encryption strategies at the pixel level. Guided by the hierarchical region map, this mechanism dynamically selects the most appropriate encryption parameters for each pixel, thereby optimizing overall system performance while maintaining security guarantees.

The core principle of strength control is the parameter mapping mechanism. Based on the pixels marked at different levels in the hierarchical map, the system automatically selects corresponding encryption parameter combinations:

(8) $$EncryptionParams(x,y)\;=\left\{\begin{array}{c} (R_{edge},M_{edge}),\;if\;Layer(x,y)\;=\;1\;\\ (R_{core},M_{core}),\;if\;Layer(x,y)\;=\;2 \end{array}\right.,$$

where R represent the number of encryption rounds and M represent the bit mask. Edge regions use fewer rounds and partial bit modification, while core regions use more rounds and complete bit modification.

The advantage of this differentiated strategy lies in reasonable resource allocation. For edge regions with lower privacy sensitivity, lightweight encryption saves computational resources. While for core regions with higher privacy sensitivity, full encryption ensures security. This allocation strategy enables the system to achieve optimal privacy protection effects under limited computational resources.

The decryption process strictly follows the reverse operation sequence of encryption. In edge regions, decryption involves reverse key mixing and reverse initial confusion. For core regions, an additional reverse circular right shift operation is required. The decryption parameters are also selected based on hierarchical map guidance, ensuring complete consistency of encryption and decryption processes.

The differentiated control mechanism is further optimized for implementation efficiency. Parameter lookup tables are precomputed to avoid redundant calculations at runtime, while vectorized operations enable batch processing of pixels with identical parameters. Additionally, utilizing spatial locality principles to optimize memory access patterns could reduce processor cache miss counts to improve data access efficiency.

### 3.4. System Architecture and Key Algorithms

#### 3.4.1. Overall System Architecture Design

**Figure 1:**
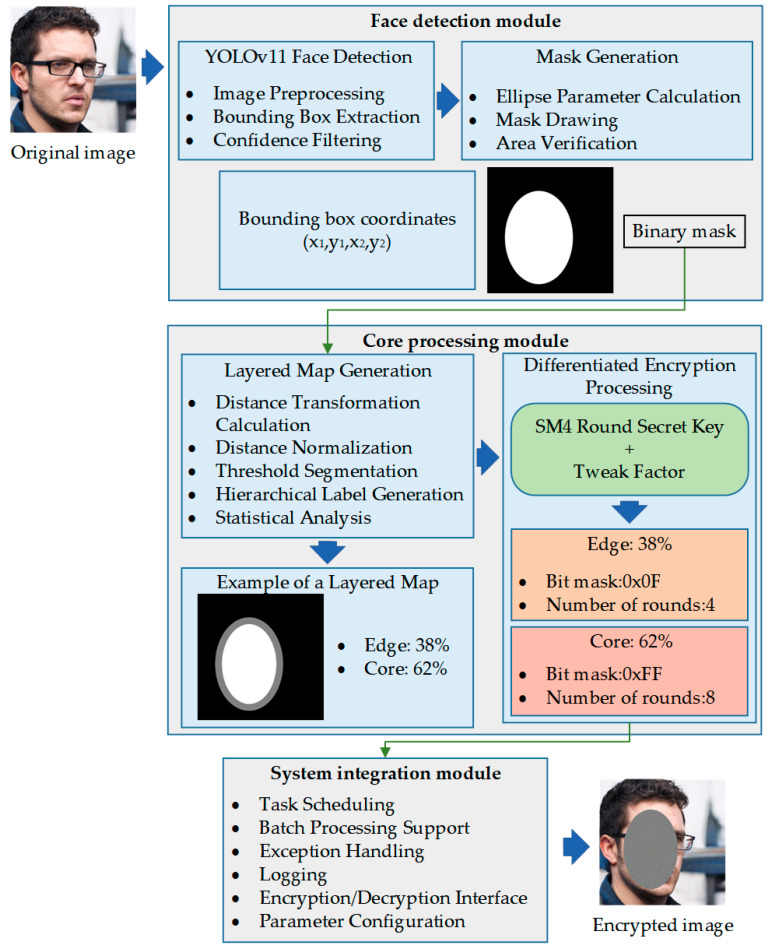
Architecture diagram of a face privacy protection system based on H-FPE.

As shown in Figure 1, this study designs a three-layer system architecture based on modular concepts. Through functional separation and interface standardization, the design achieves the goals of high cohesion and low coupling with each functional module undertaking clear responsibilities. Modules interact through well-defined interfaces, thereby ensuring the scalability, maintainability, and testability of this algorithm.

The face detection module serves as the system’s input processing layer, tasked with precisely locating facial regions from raw images. This module provides high-precision face localization capabilities through modern object detection techniques, and its output includes standardized bounding box coordinates and binary mask information, providing accurate region localization for subsequent processing. Additionally, the design of the detection module considers the requirements of different application scenarios, and it supports both single-face and multi-face detection with good robustness and adaptability.

The core processing module is the implementation layer of the system algorithm, encapsulating key technologies such as hierarchical map generation and differentiated encryption-decryption. This module internally employs highly optimized data structures and algorithm implementations, and it supports multiple performance optimization strategies. The design of the processing module fully considers computational efficiency and memory usage, which enables handling large-size images and batch data.

The system integration module serves as the coordination control layer, which is responsible for orchestrating and managing the entire processing workflow. This module provides a unified external interface, shielding the complexity of internal implementation. Additionally, the integration module undertakes system-level functions such as error handling, performance monitoring, and resource management to ensure overall system stability and reliability.

The three-layer architecture design follows the single responsibility principle, with each module focuses on a specific functional domain. Interaction between modules is achieved through standardized data formats and interface protocols, which effectively prevents tight coupling relationships. This design gives the system excellent scalability, facilitating functional upgrades and performance optimization.

#### 3.4.2. Face Detection Algorithm Based on YOLOv11

Face detection serves as the initial step of the privacy protection workflow, where its accuracy directly affects the effectiveness of subsequent encryption processing. This study selects YOLOv11 as the basic framework for face detection. This framework has the advantages of high detection accuracy, fast processing speed, and reasonable resource consumption.

YOLOv11 employs an end-to-end detection architecture that transforms object detection into a regression problem enabling simultaneous object localization and classification in a single forward propagation. This design avoids the complexity of traditional two-stage methods and significantly improves detection efficiency. Additionally, the loss function of the YOLOv11 algorithm comprehensively considers three aspects: bounding box regression, object confidence, and class classification, thereby improving object detection performance through multi-task learning.

To address the specific requirements of face detection, this study employs the YOLOv11-face model specifically optimized for faces. This model makes specific adjustments on the standard architecture, including optimization of anchor box design and adjustment of feature extraction networks. With an input resolution is set to 640 × 640 pixels, the model maintains detection accuracy while achieving balanced processing efficiency.

Face detection post-processing includes two key steps, i.e., confidence filtering and non-maximum suppression (NMS). The confidence threshold is set to 0.5 to filter low-quality detection results, while NMS is used to eliminate duplicate detection to ensure each face is represented by a single detection. These post-processing steps are important for improving the reliability of detection results.

Considering the natural shape characteristics of faces, this study employs elliptical masks instead of traditional rectangular masks. Elliptical masks can more accurately represent facial region boundaries, thereby reducing erroneous inclusion of background pixels. The mask generation process derives from the geometric parameters of detection boxes, computing the center point and semi-axis lengths of the ellipse to generate region descriptions conforming to facial contours.

#### 3.4.3. Hierarchical Map Generation Algorithm

Hierarchical map generation is the key link connecting face detection and differentiated encryption, with its quality directly affecting the effectiveness of encryption strategies. This generation algorithm converts binary facial masks into hierarchical guidance maps containing level information, thereby providing accurate spatial guidance for pixel-level differentiated processing.

This algorithm employs efficient Euclidean distance transform implementation using a separable kernel method, which decomposes two-dimensional distance computation into two one-dimensional processing operations in row and column directions, significantly reducing computational complexity. The algorithm completes distance computation through two traversals: a forward traversal establishes initial distance estimation, followed by a backward traversal that performs distance correction. Finally, this process yields a precise Euclidean distance from each pixel in the facial region to the boundary.

After distance computation is completed, the algorithm executes normalization processing to eliminate scale effects. As defined in Equation (2), the normalization process first determines the maximum distance value within the facial region, then maps all distance values to the [0, 1] interval. This processing ensures consistent performance of the algorithm across different face sizes, improving the stability of hierarchical results.

The hierarchical map is constructed by comparing normalized distance values with preset thresholds. The algorithm first creates a level identification map of the same size as the input image, then assigns level identifiers to each pixel according to the hierarchical rule defined in Equation (3). The finally generated hierarchical map encodes regional attribution with integer values: background region is 0, edge region is 1, and core region is 2.

This algorithm generates detailed statistical information, including pixel distribution for each level, area proportions, and distance characteristics. These statistical data provide important basis for system performance evaluation and parameter optimization. By analyzing statistical results, the rationality and effectiveness of hierarchical strategies can be evaluated.

In order to facilitate result verification and parameter tuning, this algorithm provides visualization functionality for hierarchical maps. This visualization employs grayscale mapping: background regions are displayed as black with grayscale value 0, edge regions are displayed as medium gray with grayscale value 128, and core regions are displayed as white with grayscale value 255. This visualization scheme can intuitively display the hierarchical structure of facial regions, facilitating understanding of algorithm behavior and optimization of parameter settings. The level identification map, statistical information, and visualization results output by the algorithm jointly ensure the accuracy of hierarchical maps, providing reliable spatial guidance for subsequent differentiated encryption processing.

## 4. Experimental Results and Analysis

This section presents a comprehensive experimental evaluation to validate the effectiveness of H-FPE. The experimental environment configuration and dataset partitioning are first presented, and the performance of our method against existing algorithms is then comparatively analyzed from two dimensions, i.e., privacy protection strength and algorithm utility. Through quantitative analysis and visual demonstrations, we demonstrate the advantages of the hierarchical encryption strategy in balancing privacy protection and utility.

### 4.1. Experimental Environment and Dataset

#### 4.1.1. Experimental Environment Configuration

The experiments in this study involve large-scale image data encryption processing and deep learning model inference computation, which impose high requirements on computational resources. To ensure efficient execution of experiments and reliability of results, the experimental platform is equipped with high-performance GPUs to accelerate deep learning model inference, along with sufficient memory and storage space to support large-scale dataset processing. Detailed configuration information is presented in Table 2.

#### 4.1.2. Dataset Selection and Preprocessing

This experiment employs the TJU-DHD dataset [33,34] and the MPII Human Pose dataset as evaluation benchmarks. The TJU-DHD dataset, a large-scale dataset specifically designed for face detection and recognition research, contains diverse facial images covering different lighting conditions, shooting angles, and image qualities, thereby comprehensively reflecting image feature variations in real application scenarios. The MPII Human Pose dataset provides rich human pose data, containing numerous high-quality images suitable for pedestrian detection tasks. The combination of these two datasets not only ensures diversity and representativeness of experimental scenarios, but also supports a complete evaluation chain from facial privacy protection to pedestrian detection utility.

According to actual application requirements and image quality characteristics in CAVs, this paper divides the dataset into three representative typical application scenarios. The first scenario (S1) comprises 3000 images containing a single-person close-range face scenario with a face height ≥ 50 pixels, belonging to the clear large face category. This scenario mainly corresponds to application tasks requiring close-range precise recognition, such as driver identity verification, fatigue monitoring, and emotion analysis. Facial regions are clearly visible with rich feature information. Therefore, privacy protection requirements are most stringent. The second scenario (S2) includes 1000 images containing a single-person long-range face scenario, but with a face height < 50 pixels, representing the small face category. This scenario typically appears in long-distance recognition tasks such as external pedestrian detection and remote security monitoring. Due to smaller facial regions and limited feature information, higher utility requirements are placed on encryption algorithms while ensuring privacy protection. The third scenario (S3) consists 5000 images containing two or more people with detectable faces. This scenario corresponds to applications such as multi-passenger monitoring inside vehicles and pedestrian detection in complex traffic scenarios. Its high complexity demands maintained detection system accuracy and stability while protecting multiple individual privacies.

### 4.2. Privacy Protection Strength Comparison Experiments

#### 4.2.1. Selection of Comparison Algorithms

To effectively evaluate the privacy protection effectiveness of our method, this paper selects two representative TPE algorithms as the benchmark. These two algorithms have different characteristics in design philosophy and technical implementation by evaluating the characteristics of the proposed method from different metrics.

The TPE algorithm operates on the core principle of frequency-domain transformation [35,36], performing encryption by converting images from the spatial domain to the frequency domain. The algorithm first performs discrete cosine transform (DCT) or wavelet transform on images to decompose the image into low-frequency and high-frequency components. Low-frequency components, which contain basic image structure and overall feature information, are preserved or undergoes only slight perturbation to maintain thumbnail visibility. In contrast, high-frequency components, containing detail texture features and identity-related recognition information, are subjected to randomization processing or scrambling operations. However, this method has inherent security defects. The separation between frequency-domain components is not completely independent, and low-frequency components still leak substantial identity information. Meanwhile, due to quantization errors and information loss in the transformation process, the algorithm cannot guarantee complete reversibility, resulting in image quality degradation after decryption.

The ideal TPE algorithm employs more advanced order-preserving encryption technology, centered around a rank-encipher mechanism [37,38]. The algorithm establishes an order-preserving mapping relationship from pixel values to encrypted values, ensuring that the relative size relationships of pixels remain unchanged before and after encryption. Specifically, the algorithm first extracts all pixel values in the image, sorts them by size, and uses keys to generate an order-preserving pseudo-random sequence, mapping original pixel values to a new value space. This mapping has strict monotonicity. If original pixel value A is greater than B, then encrypted value A is also necessarily greater than B. This approach maintains the overall statistical properties and visual structure of images while achieving a completely reversible encryption process. Although ideal TPE theoretically has stronger security because it avoids information leakage problems in frequency-domain separation of TPE, its order-preserving property also limits the degree of encryption randomness.

**Figure 2:**
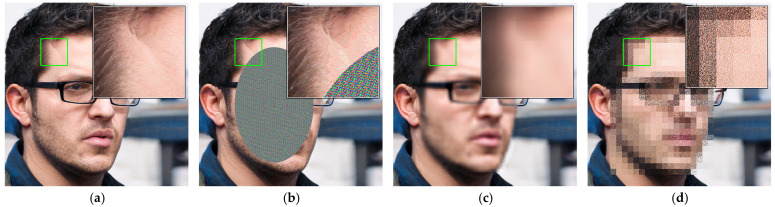
(**a**) Original image; (**b**) image encrypted by H-FPE; (**c**) image encrypted by TPE; (**d**) image encrypted by ideal TPE.

In contrast, H-FPE has fundamental differences in design philosophy. Rather than relying on frequency-domain transformation or order-preserving mapping, this method directly performs hierarchical processing in the pixel domain. By applying distance transformation, facial regions are partitioned into levels with different privacy sensitivities, and differentiated encryption strengths for each level are applied accordingly. This design both avoids the information loss problem of TPE and breaks through the order-preserving constraints of ideal TPE, enabling truly randomized encryption while guaranteeing complete reversibility. As shown in Figure 2, the encryption effects of the three algorithms on the same facial image exhibit obvious differences. Figure 2a–d present the original image, along with the encryption results of H-FPE, TPE, and ideal TPE, respectively. From visual effects, H-FPE presents stronger privacy protection, with the encrypted facial region showing no structurally discernible features.

#### 4.2.2. Evaluation Metrics

To quantify the privacy protection effects of different encryption algorithms, scientifically sound security evaluation metrics are required. Considering that the essence of facial encryption is to prevent unauthorized information extraction by increasing data randomness and unpredictability, this study employs information theory-based evaluation methods. Specifically, we adopt Shannon information entropy as the core evaluation metric, which effectively quantifies the degree of data randomness. As an important standard for evaluating encryption strength, Shannon entropy has been widely applied to security analysis of various encryption algorithms.

For an image containing *N* different pixel values, its Shannon entropy is defined as:

(9) $$H=-\sum_{i\;=\;1}^{N}p_{i}\log_{2}(p_{i}),$$

where $p_{i}$ represents the probability of pixel value i appearing in the image. For 8-bit grayscale images or single channels of RGB color images, the theoretical maximum entropy is 8 bits. This indicates that all 256 possible pixel values appear with equal probability. At this point, the data reaches a completely random state.

In order to comprehensively evaluate encryption effects, this paper also introduces the entropy efficiency metric:

(10) $$\eta =\frac{H_{encrypted}}{H_{max}}\times 100\%,$$

where $H_{encrypted}$ is the actual entropy value of the encrypted image and $H_{max}$ is the theoretical maximum entropy. Entropy efficiency reflects the percentage to which the encryption algorithm achieves the ideal randomization degree. The closer this value is to 100%, the closer the encrypted data distribution is to a completely random state, implying stronger privacy protection. Additionally, entropy increment is used to directly measure the degree to which the encryption process enhances image randomness. A positive entropy increment indicates that the encryption process enhances data randomness, whereas a negative entropy increment indicates that the encryption process instead introduces regularity and reduced unpredictability. By systematically calculating and comparing information entropy and its derived metrics of images before and after encryption, we can objectively evaluate the actual effects of different algorithms in privacy protection, thereby providing a quantitative basis for judging algorithm superiority.

#### 4.2.3. Information Entropy Analysis Results

**Figure 3:**
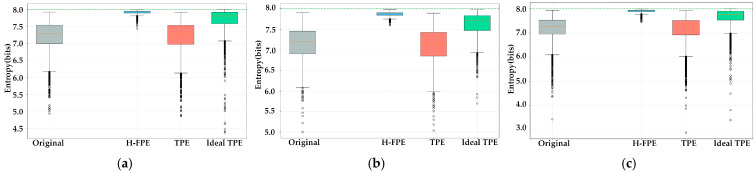
(**a**) Information entropy distribution comparison for scenario S1; (**b**) information entropy distribution comparison for scenario S2; (**c**) information entropy distribution comparison for scenario S3.

Figure 3 presents the information entropy distribution comparison of each algorithm under three typical application scenarios. As shown in Figure 3a–c, the encryption entropy value box plots of the FPE method are distinctly shifted toward the high-entropy region, with medians and quartiles all approaching the theoretical maximum value of 8 bits. This indicates that the vast majority of encrypted images have achieved extremely high randomness levels. Specifically, in the S1 scenario, the entropy values after FPE are concentrated in the 7.8–8.0 bit interval, and the narrow box indicates stable and consistent encryption effects. In contrast, the encryption entropy value box plot of TPE essentially overlaps with the original images, with only a marginal increase in median entropy, indicating inadequate encryption strength and significant retention of original information. The performance of ideal TPE is between the two encryption entropy values, show some improvement, but the median is approximately 7.6 bits. There is still a considerable gap from the theoretical maximum value. Moreover, the wide box range indicates large fluctuations in algorithm encryption effects across different images.

Table 3 presents the information entropy metric comparison of the three algorithms under different scenarios. In terms of entropy increment, H-FPE achieves entropy increases of 0.7486, 0.7697, and 0.7865 bits in the S1, S2, and S3 scenarios, respectively. The average entropy increment reaches 0.7683 bits, which is 1.71 times the average entropy increment of the ideal TPE algorithm. Specially, the entropy increment of the TPE algorithm is negative, at −0.0366, −0.0857, and −0.0512 bits, respectively, indicating that this algorithm not only fails to enhance image randomness but instead introduces regularity due to frequency-domain transformation and quantization processes, thereby reducing data unpredictability. Regarding the entropy efficiency, H-FPE achieves entropy efficiency above 98% in all three scenarios, reaching 99.0% in S1 and 98.8% in S3. This indicates that the encrypted pixel value distribution is already very close to the ideal uniform random distribution. The entropy efficiency of ideal TPE ranges between 95% and 96%, while superior to TPE, there remains room for improvement when compared to H-FPE.

**Figure 4:**
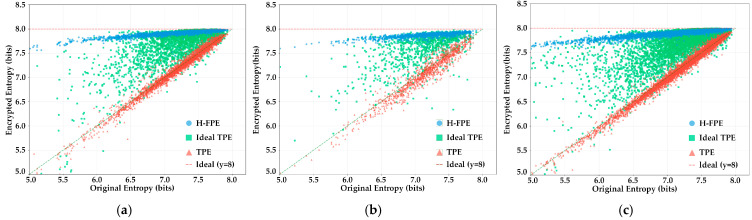
(**a**) Entropy transformation scatter comparison for scenario S1; (**b**) entropy transformation scatter comparison for scenario S2; (**c**) entropy transformation scatter comparison for scenario S3.

Figure 4 presents scatter plots of entropy transformation for each encryption algorithm from another perspective. The horizontal axis represents the entropy values of original images, while the vertical axis corresponds to the entropy values after encryption. Each data point represents the entropy value changes before and after encryption for one image. As shown in Figure 4a–c, the scatter points of H-FPE are almost entirely concentrated in a narrow region near y = 8, forming a dense band distribution close to horizontal. This pattern indicates that, regardless of how original image entropy values vary to near-ideal levels, regardless of the encryption states, demonstrating extremely strong encryption robustness. In contrast, the scatter points of the TPE algorithm are mainly distributed near the diagonal line y = x, indicating a high correlation between original and encrypted entropy and implying negligible encryption effect. The scatter point distribution of the ideal TPE algorithm presents a certain upward trend, while the distribution is relatively scattered, with most encrypted entropy values falling in the 7.4–7.8 bits, indicating incomplete realization of its encryption potential. It is noteworthy that results across the three scenarios show high consistency, further validating the stability and reliability of H-FPE in different environments.

**Figure 5:**
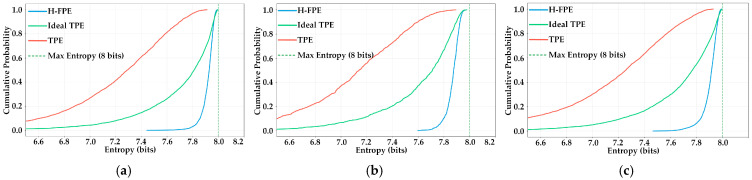
(**a**) CDF comparison of entropy values for scenario S1; (**b**) CDF comparison of entropy values for scenario S2; (**c**) CDF comparison of entropy values for scenario S3.

Figure 5 presents the cumulative distribution function (CDF) comparison of encrypted image entropy values, which intuitively reflect the image distribution proportion at different entropy levels. As shown in Figure 5a–c, the CDF curve of H-FPE rises sharply, jumping from 0 to close to nearly 1.0 near the entropy value of 7.8 bits. This indicates that almost all encrypted images have entropy values concentrated in the high-entropy interval of 7.8–8.0 bits. For instance, in scenario S1, the cumulative probability already exceeds 95% at 7.9 bits, indicating that over 95% of encrypted images achieve an extremely high security levels. In contrast, the CDF curve of TPE is relatively gentle, with the distribution range extending from 6.8 bits to 7.4 bits. The cumulative probability grows slowly and reflects the instability and inconsistency of encryption effects. Although the CDF curve of ideal TPE shows improvement compared to TPE, with a relatively concentrated distribution in the 7.5–7.8 bit interval, it still clearly lags behind our method overall. Notably, in scenario S2, characterized by lower original image quality, H-FPE still maintains excellent encryption effects. CDF curve characteristics are essentially consistent with the S1 and S3 scenarios and further proves the method’s scenario adaptability.

### 4.3. Algorithm Utility Verification Experiments

#### 4.3.1. Evaluation Metrics

To verify whether H-FPE preserves image functional utility while protecting privacy, this paper designs utility evaluation experiments based on pedestrian detection task, which is one of the core functions in CAVs. By comparing performance differences between original images and encrypted images on pedestrian detection tasks, we quantitatively evaluate the impact degree of encryption algorithms on image utility. This experiment employs the currently leading YOLOv11 model as the detector, with the confidence threshold set to 0.5 and the intersection over union (IOU) threshold set to 0.9 to judge the matching relationship between detection boxes and ground truth annotation boxes.

To comprehensively evaluate detection performance, this paper adopts the following four key metrics.

Precision is defined as the ratio of correctly detected targets to total detections. The calculation formula is:

(11) $$P=\frac{TP}{TP\;+\;FP},$$

where TP represents true positives, which is the number of correctly detected targets, and FP represents false positives, which is the number of falsely detected targets. Precision reflects the accuracy of detection results, with higher precision indicating fewer false alarms.

Recall is defined as the ratio of correctly detected targets to total actual targets, and the calculation formula is:

(12) $$R=\frac{TP}{TP\;+\;FN},$$

where FN represents false negatives (number of missed targets). Recall reflects the detector’s ability to detect all true targets. High recall means the detector rarely misses true targets.

*F*1-score is the harmonic mean of precision and recall, and can comprehensively evaluate detection performance. The calculation formula is:

(13) $$F1=\frac{2\;\times P\;\times R}{P\;+\;R},$$

Effective Detection Rate (*EDR*) is a metric specially defined in this paper. It is specifically used to measure the detection retention capability of encrypted images relative to original images. The calculation formula is:

(14) $$EDR=\frac{TP}{GT},$$

where GT represents the total number of true targets. *EDR* specifically measures the proportion of correctly matched detections among original targets.

#### 4.3.2. Detection Performance Comparison Results

Table 4 presents detailed pedestrian detection performance comparison results of the three encryption algorithms under three typical scenarios. Overall, the H-FPE method maintains detection accuracy above 97% across all tested typical application scenarios, demonstrating excellent utility retention capability. In the S1 scenario with single-person close-range face, H-FPE achieves an F1-score of 0.9907, with precision and recall are 0.9925 and 0.9906, respectively, which is only 0.0042% lower than TPE. It is noteworthy that although TPE has a slight advantage in utility metrics, combined with the previous privacy protection strength analysis, its encryption effect is almost negligible. This weak advantage in utility is obtained at the cost of sacrificing privacy protection. In contrast, the F1-score of ideal TPE is 0.9728, significantly lower than the first two algorithms. Its number of missed detections reaches 116, which is 2.15 times that of H-FPE. This indicates that the order-preserving encryption mechanism of this algorithm affects the distinguishability of image features to a certain extent.

In the S2 scenario, where smaller facial regions and limited feature information substantially increase detection difficulty, H-FPE demonstrates strong robustness. The F1-score reaches 0.9797, only 0.0154 lower than TPE. In contrast, the performance of the ideal TPE method drops substantially, and the F1-score falls to 0.9097, a difference of 7% from H-FPE. The number of missed detections is as high as 117, which indicates that the order-preserving property of ideal TPE has obvious limitations when processing small targets. In comparison, the hierarchical encryption strategy effectively retains key features such as facial contours by adopting lightweight encryption for edge regions, thereby achieving a more effective balance between privacy protection and utility.

In the S3 scenario, the detection system needs to simultaneously process multiple targets, necessitating higher requirements in terms of algorithm stability. Experimental results show that H-FPE continues to maintain stable performance, successfully detecting 28,011 among 28,320 true targets, with an effective detection rate of 98.87%. The performance ranking of the three algorithms in this scenario remains consistent with the previous two scenarios, further validating the reliability of experimental conclusions. Across all the three scenarios, the difference between proposed method and TPE in utility metrics averages only 0.5 percentage points. However, the advantage in privacy protection strength achieves a qualitative leap. This outcome strongly demonstrates the effectiveness of the hierarchical encryption strategy.

**Figure 6:**
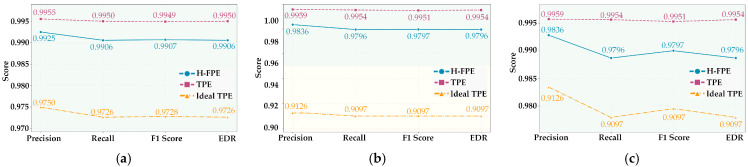
(**a**) Performance metric trend comparison for scenario S1; (**b**) performance metric trend comparison for scenario S2; (**c**) performance metric trend comparison for scenario S3.

Figure 6 intuitively displays the comparison results of the three algorithms on four key performance metrics in the form of line charts. As shown in Figure 6a–c, the performance curves of H-FPE and TPE remain very close across the three scenarios. The differences between the two in the four dimensions of precision, recall, F1-score, and effective detection rate are all extremely small. For instance, in the S1 scenario, the line charts for the four metrics are distributed almost parallel in the high-level interval near 0.99, highlighting the similarity of the two methods in utility retention. In contrast, the performance curve of the ideal TPE method is significantly lower than the first two. Particularly in the S2 scenario, where all its metrics all fall around 0.91, resulting in approximately 8% performance gap compared to H-FPE.

**Figure 7:**
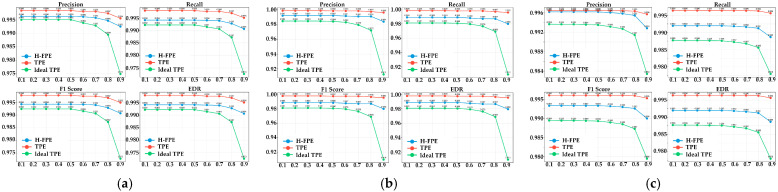
(**a**) IOU threshold sensitivity comparison in scenario S1; (**b**) IOU threshold sensitivity comparison in scenario S2; (**c**) IOU threshold sensitivity comparison in scenario S3.

Figure 7 illustrates the change trends of detection performance for the three encryption algorithms under different IOU thresholds. This analysis has important significance for evaluating the parameter robustness of encryption algorithms. The IOU threshold increases gradually from 0.1 to 0.9, indicating that requirements for detection box localization accuracy become increasingly stringent. As shown in Figure 7a–c, H-FPE demonstrates excellent stability at each IOU threshold. Four performance curves corresponding to precision, recall, F1-score, and effective detection rate show a gentle declining trend as the IOU threshold increases, indicating that even under high IOU requirements, this method can still maintain relatively high detection accuracy. For instance, in the S1 scenario, when the IOU threshold increases from 0.1 to 0.9, the F1-score of the H-FPE method decreases from 0.994 to 0.991. The decline magnitude is only 0.3%.

In contrast, the ideal TPE algorithm exhibits relatively high sensitivity to IOU threshold changes. In the S2 scenario, when the IOU threshold is set to 0.9, the recall rate of ideal TPE drops sharply from 0.969 under the 0.8 threshold to 0.91, with a decline of 5.9%. This indicates that while images encrypted by ideal TPE can still be recognized by detectors under low precision requirements, they struggle to meet stricter accuracy requirements. The underlying reason is that although order-preserving encryption maintains pixel value ordering, it alters the actual pixel distribution, leading to feature extraction deviations that impair precise bounding box localization. Meanwhile, TPE method maintains optimal performance at each IOU threshold, though this is achieved at the cost of providing almost no privacy protection.

### 4.4. Algorithm Performance Analysis

#### 4.4.1. Evaluation Metrics

In order to verify the actual deployment requirements of H-FPE in terms of real-time performance and resource consumption, this study introduced comprehensive performance evaluation indicators covering time performance, throughput capacity and resource utilization. Through these indicators, we can comprehensively assess the performance of H-FPE compared to ideal TPE and ideal TPE.

In order to evaluate the encryption performance of the algorithm itself, this paper introduces the core algorithm time $T_{core}$. $T_{core}$ represents the time consumed only during the encryption or decryption operations, excluding the cost of face detection. For a dataset containing *n* images, the calculation formula is:

(15) $$T_{core}=\frac{1}{n}\sum_{i\;=\;1}^{n}t_{i}^{core}$$

where $t_{i}^{core}$ represents the core processing time for the i-th image.

Meanwhile, this paper calculates the time $T_{detect}$ spent on face detection throughout the entire encryption process. The calculation formula is:

(16) $$T_{detect}=\frac{1}{n}\sum_{i\;=\;1}^{n}t_{i}^{detect}$$

where $t_{i}^{detect}$ represents the detection time for the i-th image. This metric is used to quantify the algorithm’s overhead relative to the detection baseline.

The end-to-end total delay $T_{total}$ is defined as the complete processing time from the input image to the encrypted output. The calculation formula is:

(17) $$T_{total}=T_{detect}+T_{core}+T_{overhead}$$

where $T_{overhead}$ represents system overhead. This metric reflects the response time of the complete encryption system when deployed in practice.

Throughput F is defined as the number of frames processed per second. The calculation formula is:

(18) $$F=\frac{1000}{T_{total}}$$

To analyze the computational complexity of the three algorithms, we conduct the calculation from two perspectives: time complexity and space complexity. Assume that the face region consists of *N* pixels, and each pixel has *B* possible values. The number of encryption rounds is *R*.

H-FPE performs *R* rounds of Feistel network operations on each pixel of the face region, and the computational complexity of each round is O(1). The time complexity of H-FPE is linearly related to the number of pixels in the image and the number of encryption rounds. Therefore, the time complexity of H-FPE is O(N × R). The space complexity of H-FPE only requires storing the input image and intermediate states, and is linearly related to the number of pixels in the image. The space complexity of H-FPE is O(N).

Ideal TPE needs to construct a monotonic mapping table to maintain the relative order of pixel values. For each pixel value, a sorting operation needs to be performed to determine its position in the ordered sequence. Therefore, the overall time complexity of ideal TPE is O(N × B × logB) and the space complexity is $O(N\;+\;B^{2})$.

TPE is encrypted based on DCT. The computational complexity of DCT transformation is $O(B^{2})$. For N pixels in the face area, the DCT transformation is performed separately, and the overall time complexity is $O(N\;\times \;B^{2})$. The space complexity of TPE is O(N) because the encryption process only requires the storage of the input image and the transformation coefficients.

#### 4.4.2. Experimental Results of Algorithm Performance Comparison

To evaluate the computational performance of the three algorithms, we randomly selected 100 facial images for testing. Each measurement was repeated multiple times to ensure the reliability of the statistics.

Table 5 presents the complete performance indicators of the three algorithms. From the perspective of operational efficiency, the average encryption time of H-FPE is 462.4 ms, which is 1.28 times faster than ideal TPE and 4.22 times faster than TPE. This performance advantage stems from the linear time complexity of H-FPE. After decomposing the end-to-end processing time, we found that face detection accounted for 8.4% and 7.2% of the total encryption delay for H-FPE and ideal TPE, respectively. This indicates that the core encryption algorithm is the main computational bottleneck. Additionally, although the face detection time of TPE accounts for only 1.5%, it requires additional DCT preprocessing, resulting in a total processing time of 31.5% for detection and preprocessing. From the perspective of memory usage, the memory overhead of the three methods remains at a reasonable level. The memory overhead of H-FPE is slightly higher, mainly used for storing the intermediate states of hierarchical encryption, but the difference compared to ideal TPE and TPE is less than 10%, confirming its suitability for resource-constrained vehicle environments.

Table 6 quantifies the performance advantages of H-FPE over the two comparison algorithms. H-FPE outperforms ideal TPE and TPE by achieving 1.28 times and 4.22 times encryption acceleration, respectively. The decryption speed of H-FPE is 1.32 times faster than that of ideal TPE. The encryption throughput of H-FPE is 12.7% higher than that of ideal TPE and 254.3% higher than that of TPE. The decryption throughput of H-FPE is 32.6% higher than that of ideal TPE, providing better performance for scenarios requiring real-time decryption access.

## 5. Conclusions

Facial data collected by autonomous sensors faces increasingly severe privacy leakage risks with the widespread application of facial recognition technology. Existing encryption methods struggle to balance privacy protection and data utility, either compromising image formats or causing system compatibility issues. To address these limitations, this paper proposes an H-FPE method which partitions facial regions into edge zones and core zones through distance transformation, applying lightweight encryption with low-bit modification for edge zones to preserve contour features and full-bit encryption for core zones to ensure privacy security. In addition, the Feistel encryption network constructed based on the SM4 algorithm guarantees complete reversibility and RGB value domain constraints, achieving format compatibility.

Experiments on the combined TJU-DHD and MPII Human Pose datasets validate the effectiveness of the method. Evaluations of privacy protection strength demonstrate that H-FPE achieves an average entropy increment of 0.7683 bits in three scenarios, with entropy efficiency being above 98%, significantly outperforming both TPE and ideal TPE. In utility evaluation, the encrypted images maintain F1-scores above 97% on pedestrian detection tasks. The performance gap with TPE is only 0.5%, yet privacy protection strength improves by approximately 10%. Furthermore, the IOU threshold sensitivity analysis proves the robustness under different parameter settings. Computational performance evaluation demonstrates that H-FPE is 1.28 times and 1.32 times faster than ideal TPE for encryption and decryption, respectively, with throughput improvements of 12.7% and 32.6%, confirming suitability for real-time CAV applications.

While the proposed method has achieved significant results, several limitations warrant further consideration. These include relatively high computational complexity, experience-dependent parameter selection, and insufficient validation against complex attack models. Future work will focus on algorithm optimization, learning-based adaptive parameter adjustment mechanisms, utility validation for multi-task scenarios, and evaluation of defense capabilities against deep learning attacks.

## Figures and Tables

**Table 1:** Comparison of facial privacy protection methods.

Approach	Format Preservation	Reversibility	Task Utility	Computational Cost
Anonymization	Partial (blur/pixelation)	No	Low	Low
Adversarial Perturbation	High (minimal distortion)	Not required	Medium-High	Low
Encryption-based	Full (FPE/ideal TPE) or None (AES)	Yes	Low-Medium	Low-Very High
Generative Models	High (synthetic faces)	No	High	Very High
H-FPE	Full (pixel-level)	Yes (lossless)	High	Low

**Table 2:** Experimental environment hardware configuration information.

Experimental Environment	Configuration
CPU	13th Gen Intel(R) Core(TM) i9-13900KF 3.00 GHz
GPU	NVIDIA GeForce RTX 4090D
Memory	32 G × 2
Storage	4TB 7.2K RPM
Operating System	Windows 10 Pro for Workstations
Computational Platform	CUDA 11.6
Development Language	Python 3.9.23
Deep Learning Framework	PyTorch 1.12.1

**Table 3:** Information entropy comparison results of three algorithms.

Scenario	Algorithm	Original Avg Entropy (Bits)	Encrypted Avg Entropy (Bits)	Entropy Increase (Bits)	Entropy Efficiency (%)
S1	H-FPE	7.1682	7.9168	0.7486	99.0
	Ideal_TPE	7.2393	7.6908	0.4515	96.1
	TPE	7.2415	7.2048	−0.0366	90.1
S2	H-FPE	7.0981	7.8678	0.7697	98.3
	Ideal_TPE	7.1659	7.6000	0.4341	95.0
	TPE	7.1725	7.0868	−0.0857	88.6
S3	H-FPE	7.1171	7.9035	0.7865	98.8
	Ideal_TPE	7.1966	7.6608	0.4642	95.8
	TPE	7.1987	7.1475	−0.0512	89.3

**Table 4:** Pedestrian detection performance comparison results of three algorithms in typical application scenarios.

Scenario	Algorithm	Precision	Recall	F1-Score	Effective Detection Rate	Correct Matches
S1	H-FPE	0.9925	0.9906	0.9907	0.9906	3142
	Ideal_TPE	0.9750	0.9726	0.9728	0.9726	3080
	TPE	0.9955	0.9950	0.9949	0.9950	3174
S2	H-FPE	0.9836	0.9796	0.9797	0.9796	1061
	Ideal_TPE	0.9126	0.9097	0.9097	0.9097	983
	TPE	0.9959	0.9954	0.9951	0.9954	1090
S3	H-FPE	0.9928	0.9887	0.99	0.9887	28,011
	Ideal_TPE	0.9834	0.9779	0.9795	0.9779	27,714
	TPE	0.9957	0.9956	0.9953	0.9956	28,204

**Table 5:** End-to-end performance indicator comparison.

Algorithm	Operation	Detection Time (ms)	Core Algorithm Time (ms)	Total Latency (ms)	Throughput (FPS)
H-FPE	Encrypt	68.3	462.4	809	1.24
	Decrypt	-	439.3	439.3	2.28
Ideal TPE	Encrypt	65.07	591.7	905.2	1.1
	Decrypt	-	579.9	579.9	1.72
TPE	Encrypt	43	1950.2	2847.4	0.35

**Table 6:** Relative performance analysis.

Metric	H-FPE vs. Ideal TPE	H-FPE vs. TPE
Encryption Speedup	1.28×	4.22×
Decryption Speedup	1.32×	TPE cannot be decrypted
Encryption throughput improvement	+12.7%	+254.3%
Decryption throughput improvement	+32.6%	TPE cannot be decrypted

## Data Availability

The data presented in this study are from the following openly available datasets: the TJU-DHD dataset and the MPII Human Pose dataset. The TJU-DHD dataset can be accessed via its official repository, https://github.com/tjubiit/TJU-DHD?tab=readme-ov-file#3 (accessed on 15 August 2025). The MPII Human Pose dataset can be accessed via its official repository, https://www.mpi-inf.mpg.de/departments/computer-vision-and-machine-learning/software-and-datasets/mpii-human-pose-dataset#download (accessed on 15 August 2025).

## Abbreviations

The following abbreviations are used in this manuscript:

CAVs	Connected Automated Vehicles
H-FPE	Hierarchical Format-preserving Encryption
FPE	Format-Preserving Encryption
TPE	Thumbnail-Preserving Encryption
ITS	Intelligent Transportation Systems
NMS	Non-Maximum Suppression
ROI	Region of Interest
AES	Advanced Encryption Standard
GANs	Generative Adversarial Networks
VAEs	Variational Autoencoders
CIAGAN	Conditional Identity Anonymization Generative Adversarial Network
AAM	Active Appearance Model
MI-FGSM	Momentum Iterative Fast Gradient Sign Method
RLWE	Ring Learning with Error
LSB	Least Significant Bit
DCT	Discrete Cosine Transform
3D	Three-Dimensional
IOU	Intersection over Union
EDR	Effective Detection Rate
CDF	Cumulative Distribution Function

## References

[1] Fan J., Fan L., Ni Q., Wang J., Liu Y., Li R., Wang Y., Wang S. (2024). Perception and planning of intelligent vehicles based on BEV in extreme Off-Road scenarios. IEEE Trans. Intell. Veh..

[2] Falaschetti L., Manoni L., Palma L., Pierleoni P., Turchetti C. (2024). Embedded real-time vehicle and pedestrian detection using a compressed tiny YOLO v3 architecture. IEEE Trans. Intell. Transp. Syst..

[3] Irfan M.S., Dasgupta S., Rahman M. (2024). Toward transportation digital twin systems for traffic safety and mobility: A review. IEEE Internet Things J..

[4] Chen X., Yin J., Tang K., Tian Y., Sun J. (2022). Vehicle trajectory reconstruction at signalized intersections under connected and automated vehicle environment. IEEE Trans. Intell. Transp. Syst..

[5] Zhao J., Yang X., Zhang C. (2024). Vehicle trajectory reconstruction for intersections: An integrated wavelet transform and Savitzky-Golay filter approach. Transp. A.

[6] Zhang Y., Ji J., Wen W., Zhu Y., Xia Z., Weng J. (2024). Understanding visual privacy protection: A generalized framework with an instance on facial privacy. IEEE Trans. Inf. Forensics Secur..

[7] Hellmann F., Mertes S., Benouis M., Hustinx A., Hsieh T.-C., Conati C., Krawitz P., André E. (2024). GANonymization: A GAN-based face anonymization framework for preserving emotional expressions. ACM Trans. Multimed. Comput. Commun. Appl..

[8] Caruccio L., Desiato D., Polese G., Tortora G., Zannone N. (2022). A decision-support framework for data anonymization with application to machine learning processes. Inf. Sci..

[9] Zhang X., Wang T., Ji J., Zhang Y., Lan R. (2025). Privacy-preserving face attribute classification via differential privacy. Neurocomputing.

[10] Wang T., Lin T., Liu Z., Xie X., Yao S. (2026). ADPGAN: Adaptive differential privacy-preserving GAN for image privacy. Inf. Fusion.

[11] Yuan J., Liu W., Shi J., Li Q. (2025). Approximate homomorphic encryption based privacy-preserving machine learning: A survey. Artif. Intell. Rev..

[12] Son W., Kwon S., Lee J.H. (2025). Homomorphic encryption-based facial recognition for dual-layer similarity analysis of modified images. IEEE Access.

[13] Teng L., Du L., Leng Z., Wang X. (2024). Chaotic image encryption based on partial face recognition and DNA diffusion. Appl. Intell..

[14] Zhao R., Zhang Y., Wang T., Wen W., Xiang Y., Cao X. (2025). Visual content privacy protection: A survey. ACM Comput. Surv..

[15] Chai X., Long G., Gan Z., Zhang Y. (2024). TPE-MM: Thumbnail preserving encryption scheme based on Markov model for JPEG images. Appl. Intell..

[16] Li M., Cui Q., Wang X., Zhang Y., Xiang Y. (2024). FTPE-BC: Fast thumbnail-preserving image encryption using block-churning. Expert Syst. Appl..

[17] Newton E.M., Sweeney L., Malin B. (2005). Preserving privacy by de-identifying face images. IEEE Trans. Knowl. Data Eng..

[18] Sweeney L. (2002). K-anonymity: A model for protecting privacy. Int. J. Uncertain. Fuzziness Knowl.-Based Syst..

[19] Gross R., Sweeney L., De la Torre F., Baker S. Model-Based Face De-Identification. Proceedings of the 2006 Conference on CVPRW’06.

[20] Meng L., Sun Z., Ariyaeeinia A., Bennett K.L. Retaining Expressions on De-Identified Faces. Proceedings of the 2014 37th International Convention on Information and Communication Technology, Electronics and Microelectronics.

[21] Gross R., Airoldi E., Malin B., Sweeney L., Martin D., Danezis D. (2006). Integrating Utility into Face De-Identification. Lecture Notes in Computer Science, Proceedings of the 5th International Workshop on Privacy Enhancing Technologies, Cavtat, Croatia, 30 May–1 June 2005.

[22] Sharif M., Bhagavatula S., Bauer L., Reiter M.K. Accessorize to a Crime: Real and Stealthy Attacks on State-of-the-Art Face Recognition. Proceedings of the 2016 ACM SIGSAC Conference on Computer and Communications Security.

[23] Komkov S., Petiushko A. AdvHat: Real-World Adversarial Attack on arcFace Face ID System. Proceedings of the 2020 25th International Conference on Pattern Recognition.

[24] Dong Y., Liao F., Pang T., Su H., Zhu J., Hu X., Li J. Boosting Adversarial Attacks with Momentum. Proceedings of the IEEE Conference on Computer Vision and Pattern Recognition.

[25] Li Y., Long C., Wei J., Li J., Yang F. (2023). Face Recognition Privacy Protection Method Based on Homomorphic Encryption. Inf. Secur. Res..

[26] Alsafyani M., Alhomayani F., Alsuwat H., Alsuwat E. (2023). Face image encryption based on feature with optimization using secure crypto general adversarial neural network and optical chaotic map. Sensors.

[27] Zhang Z., Cao Y., Jahanshahi H., Mou J. (2023). Chaotic color multi-image compression-encryption/LSB data type steganography scheme for NFT transaction security. J. King Saud Univ. Comput. Inf. Sci..

[28] Çiftçi S., Akyüz A.O., Ebrahimi T. (2018). A reliable and reversible image privacy protection based on false colors. IEEE Trans. Multimed..

[29] Ji H., Tian L., Wang J., Yao Y., Wang J. (2025). Face desensitization for autonomous driving based on identity de-identification of generative adversarial networks. Electronics.

[30] Sun Q., Ma L., Oh S.J., Van Gool L., Schiele B., Fritz M. Natural and Effective Obfuscation by Head Inpainting. Proceedings of the IEEE Conference on Computer Vision and Pattern Recognition.

[31] Li T., Lin L. AnonymousNet: Natural Face De-Identification with Measurable Privacy. Proceedings of the 2019 IEEE/CVF Conference on Computer Vision and Pattern Recognition Workshops.

[32] Maximov M., Elezi I., Leal-Taixé L. CIAGAN: Conditional Identity Anonymization Generative Adversarial Networks. Proceedings of the 2020 IEEE/CVF Conference on Computer Vision and Pattern Recognition.

[33] Pang Y., Cao J., Li Y., Xie J., Sun H., Gong J. (2021). TJU-DHD: A diverse high-resolution dataset for object detection. IEEE Trans. Image Process.

[34] Cao J., Pang Y., Xie J., Khan F.S., Shao L. (2022). From handcrafted to deep features for pedestrian detection: A survey. IEEE Trans. Pattern Anal. Mach. Intell..

[35] Zhang Y., Zhou W., Zhao R., Zhang X., Cao X. (2023). F-TPE: Flexible thumbnail-preserving encryption based on multi-pixel sum-preserving encryption. IEEE Trans. Multimed..

[36] Zhao R., Zhang Y., Wen W., Lan R., Xiang Y. (2024). E-TPE: Efficient thumbnail-preserving encryption for privacy protection in visual sensor networks. ACM Trans. Sen. Netw..

[37] Yuan Y., He H., Amirpour H., Qu L., Timmerer C., Chen F. (2024). IoT privacy protection: JPEG-TPE with lower file size expansion and lossless decryption. IEEE Internet Things J..

[38] Xie D., Zhang Y., Hu Z., Wang T., Chen F., Hu P. (2024). Detect-TPE: A new framework for ideal thumbnail-preserving encryption via face detection. IEEE Internet Things J..

